# Biosensor-based growth-coupling as an evolutionary strategy to improve heme export in *Corynebacterium glutamicum*

**DOI:** 10.1186/s12934-024-02556-1

**Published:** 2024-10-14

**Authors:** Aileen Krüger, Janik Göddecke, Michael Osthege, Luis Navratil, Ulrike Weber, Marco Oldiges, Julia Frunzke

**Affiliations:** Institute for Bio- and Geosciences 1, Forschungszentrum Jülich GmbH, IBG-1, 52425 Jülich, Germany

**Keywords:** Biosensor, Heme, Growth-coupling, ALE, HrtBA, ChrSA, CydD

## Abstract

The iron-containing porphyrin heme is of high interest for the food industry for the production of artificial meat as well as for medical applications. Recently, the biotechnological platform strain *Corynebacterium glutamicum* has emerged as a promising host for animal-free heme production. Beyond engineering of complex heme biosynthetic pathways, improving heme export offers significant yet untapped potential for enhancing production strains. In this study, a growth-coupled biosensor was designed to impose a selection pressure on the increased expression of the *hrtBA* operon encoding an ABC-type heme exporter in *C. glutamicum*. For this purpose, the promoter region of the growth-regulating genes *pfkA* (phosphofructokinase) and *aceE* (pyruvate dehydrogenase) was replaced with that of P_*hrtB*_, creating biosensor strains with a selection pressure for *hrtBA* activation. Resulting sensor strains were used for plate-based selections and for a repetitive batch f(luorescent)ALE using a fully automated laboratory platform. Genome sequencing of isolated clones featuring increased *hrtBA* expression revealed three distinct mutational hotspots: (i) *chrS*, (ii) *chrA*, and (iii) *cydD*. Mutations in the genes of the ChrSA two-component system, which regulates *hrtBA* in response to heme levels, were identified as a promising target to enhance export activity. Furthermore, causal mutations within *cydD*, encoding an ABC-transporter essential for cytochrome *bd* oxidase assembly, were confirmed by the construction of a deletion mutant. Reversely engineered strains showed strongly increased *hrtBA* expression as well as increased cellular heme levels. These results further support the proposed role of CydDC as a heme transporter in bacteria. Mutations identified in this study therefore underline the potential of biosensor-based growth coupling and provide promising engineering targets to improve microbial heme production.

**Supplementary Information:**

The online version contains supplementary material available at 10.1186/s12934-024-02556-1.

## Introduction

The iron-containing porphyrin heme serves as prosthetic group in essential proteins and plays a central role in many important cellular processes, like respiration and electron transfer. Additionally, heme serves as iron resource in iron-depleted environments [1–3]. Beyond its crucial role in cellular processes, heme holds significant potential in the medical and food sectors, where interest is rapidly growing. For instance, it is utilized as a supplement to treat anemia, particularly during pregnancy, due to its superior tolerability and bioavailability compared to other forms of iron [4, 5]. Moreover, heme is important as natural food coloring pigment [6] as well as a meat-flavoring agent in plant-based meat alternatives [7, 8]. Traditional heme production methods are based on extraction from animal blood via enzymatic hydrolysis. However, this process is time-intensive, expensive and with low-yield [9], as well as controversial due to the use of animal materials. Consequently, several microbial organisms were engineered for an alternative high-yield heme production [10], including studies focusing on *Saccharomyces cerevisiae* [11], *Escherichia coli* [12] and *Corynebacterium glutamicum* [13]. Since heme biosynthetic pathways are complex, intricately regulated and involve multiple steps, they offer numerous opportunities for engineering towards a high heme production [14–17]. High heme yields were recently obtained for engineered *C. glutamicum* strains, with a maximum titer of 309.18 ± 16.43 mg/L [13]. Similarly, another *C. glutamicum* strain synthesizing a total of 1.6 g/L iron-containing porphyrin derivatives was established [18]. The Gram-positive soil bacterium *C. glutamicum* is an appealing host for heme production due to its use for the industrial production of GRAS food and feed additives [19]. To date, heme production efficiencies achieved in *C. glutamicum* are certainly remarkable. However, recent studies revealed significant constraints with heme export that needed to be optimized to further enhance heme productivity, with heme secretion rates reported at approximately 45.5% [18] and 78.6% [13]. Although the latter study already demonstrated high heme secretion levels, this is achieved by a plasmid-based overexpression of *hrtBA*. For industrial production strains, the utilization of plasmids and antibiotics is not favorable and alternative approaches to achieve overexpression of *hrtBA* are certainly of interest.

The main route for heme export in *C. glutamicum* is the heme exporter HrtBA. This ABC-type transport system is tightly regulated by the specific heme-responsive two-component system (TCS) ChrSA, with ChrS being the histidine kinase and ChrA the response regulator (RR), respectively [20, 21]. The paralogous, heme-responsive TCS HrrSA, which is a global heme-responsive TCS regulating more than 200 targets, was shown to additionally influence *hrtBA* activation [22]. HrtBA activity was confirmed as crucial factor to counteract toxic heme levels by direct outward transfer, and increasing its activity further was recently shown to confer a significant increase in heme tolerance (> 100 µM) [23]. Biochemical studies and crystal structures of the closely related *Corynebacterium diphtheriae* showed that heme is indeed the substrate actively exported by HrtBA [24]. Recent advances have been made to synthetically overexpress *hrtBA* with the intention to increase heme secretion, but the overexpression of membrane transport proteins always bears a risk to alter e.g. membrane integrity or cell viability and therefore frequently has negative effects on cell growth [13, 25, 26]. Similarly, research on heme tolerance indicated that a plasmid-based overexpression of *hrtBA* even leads to severe growth defects [23].

The goal of this study was to identify mutations that lead to increased *hrtBA* expression without negatively impacting bacterial growth. For this purpose, we constructed growth-coupled biosensors, which confer a selection pressure towards elevated *hrtB* expression. To achieve this, we made use of growth-regulating genes *pfkA* and *aceE* [27], replacing the original promoter region by P_*hrtB*_, which is activated by the RR ChrA in the presence of heme. As a result, the proliferation of these strains depended on enhanced *hrtBA* promoter activity, which is triggered by increased heme availability in the cytoplasmic membrane. Plate-based selection as well as liquid, automated adaptive laboratory evolution (ALE) approaches led to the selection of strains with increased *hrtB* expression. Genome sequencing resulted in the identification of several novel mutations in *chrS* and *chrA* of the heme-responsive TCS as well as in the *cydD* gene encoding an ABC transporter essential for the cytochrome *bd* oxidase assembly. Deletion of *cydD* was shown to lead to enhanced total heme levels and is therefore corroborating the suggested role of CydD in heme export [28]. These results emphasize the potential of growth-coupled biosensor approaches for the identification of novel targets for metabolic engineering to improve microbial heme production processes.

## Materials and methods

### Bacterial strains and growth conditions

Bacterial strains used within this study are listed in Table 1. For standard cultivations, *C. glutamicum* ATCC 13032 and derivatives were streaked from glycerol stocks on agar plates containing brain heart infusion (BHI) (Difco, BD, Heidelberg, Germany) (37 g/L) and incubated at 30 °C. Single colonies were picked and incubated for ~ 8 h at 30 °C in 5 mL BHI reaction tubes, 170 rpm (for cultivation in shake flasks), or in 1 mL BHI in deep-well plates covered with breathable rayon film (VWR International, PA, United States), 900 rpm (for microtiter cultivation). This first pre-culture was used to further inoculate the second one 1:10 in CGXII minimal medium [29] (1 g/L K_2_HPO_4_, 1 g/L KH_2_PO_4_, 5 g/L urea, 42 g/L MOPS, 13.25 mg/L CaCl_2_ ⋅ 2 H_2_O, 0.25 g/L MgSO_4_ ⋅ 7 H_2_O, 10 mg/L FeSO_4_ ⋅ 7 H_2_O, 10 mg/L MnSO_4_ ⋅ H_2_O, 0.02 mg/L NiCl_2_ ⋅ 6 H_2_O, 0.313 mg/L CuSO_4_ ⋅ 5 H_2_O, 1 mg/L ZnSO_4_ ⋅ 7 H_2_O, 0.2 mg/L biotin, 30 mg/L 3,4-dihydroxybenzoate (PCA), 20 g/L D-glucose, pH 7.0). Cultivation was performed at 900 rpm (1 mL in deep-well plates) over night at 30 °C. For the main culture, a starting OD_600_ of 1 was employed in CGXII medium with adjustments as stated throughout the text, respectively. For cultivations with hemin (Sigma-Aldrich, St. Louis, United States) (stock of 2.5 mM in 20 mM NaOH, sterile filtered), further referred to as heme, the second pre-culture was performed without addition of any iron source, i.e. no FeSO_4_ and no heme, while the main culture was devoid of FeSO_4_ and the indicated amount of heme was added to the medium. If appropriate, 25 µg/mL kanamycin was added. Main cultures were performed in shaking flasks, or for online monitoring of growth and fluorescence in 48-well microtiter Flower Plates in a BioLector I or BioLector II microbioreactor cultivation system (Beckman Coulter GmbH, Aachen, Germany) [30]. The FlowerPlates were sealed with a gas-permeable sealing foil (VWR, Radnor, United States, Cat. No. 60941-086). In the microbioreactor cultivation system, backscatter (a.u.) was measured in 30 min (BioLector I) or 5 min (BioLector II) intervals as scattered light at λ: 620 nm (signal gain: 20), while YFP-fluorescence was measured at λex: 508 nm / λem: 532 nm (signal gain: 80). Specific fluorescence (a.u.) was calculated by dividing the YFP-signal by the backscatter signal for each measurement.

For cloning purposes, *Escherichia coli* strains were cultivated from a glycerol stock in Lysogeny Broth (10 g/L tryptone, 5 g/L yeast extract, 10 g/L NaCl) medium at 37 °C in a rotary shaker in shaking flasks and if needed for selection, 50 µg/mL kanamycin was added to the medium.

**Table 1:** Bacterial strains used in this study

Strain	Characteristics	Reference
*C. glutamicum* ATCC 13032	Wild type (WT), biotin auxotroph	Kinoshita, Udaka [31]
*C. glutamicum*::P_*hrtB*__*pfkA*	Integrated *hrtB*-sensor construct (terminator, *chrS-hrtB* intergenic region and first 30 bp of *hrtB* followed by stop codon, RBS and linker) upstream of *pfkA*	This work
*C. glutamicum*::P_*hrtB*__*aceE*	Integrated *hrtB*-sensor construct (terminator, *chrS-hrtB* intergenic region and first 30 bp of *hrtB* followed by stop codon, RBS and linker) upstream of *aceE*	This work
*C. glutamicum*::P_*hrtB*__*pgi*	Integrated *hrtB*-sensor construct (terminator, *chrS-hrtB* intergenic region and first 30 bp of *hrtB* followed by stop codon, RBS and linker) upstream of *pgi*	This work
*C. glutamicum*::P_*hrtB*__*hisD*	Integrated *hrtB*-sensor construct (terminator, *chrS-hrtB* intergenic region and first 30 bp of *hrtB* followed by stop codon, RBS and linker) upstream of *hisD*	This work
*C. glutamicum* Δ*cydD*	Deletion of *cydD*	This work
*C. glutamicum chrS*-Ala245fs	730delG in *chrS* leading to frameshift at Ala245, pseudokinase variant	Krüger and Frunzke [23]
*E. coli* DH5α	F^−^ ϕ80*lacZ*ΔM15 Δ(*lacZYA*-*argF*)U169 *recA*1 *endA*1 *hsdR*17(r_K_^−^ m_K_^+^) *phoA supE*44 *thi*-1 *gyrA*96 *relA*1 λ^−^; for general cloning purposes	Invitrogen

### Recombinant DNA work

Standard molecular methods were performed as previously described [32]. Plasmids were constructed by amplifying DNA fragments via PCR using the respective oligonucleotides as listed in Table S1 and enzymatically ligated into a pre-cut vector backbone using Gibson assembly [33]. Synthesis of oligonucleotides as well as Sanger sequencing for plasmid verification were performed by Eurofins Genomics (Ebersberg, Germany).

Deletion of genes in the genome of *C. glutamicum* was achieved using the suicide vector pK19*-mobsacB* [34]. Transformation of electrocompetent *C. glutamicum* cells with the respective isolated plasmids (Table 2) was performed via electroporation [35]. First and second recombination events were consequently performed and verified as described in previous studies [36]. Deletions were confirmed by amplification and Sanger sequencing.

**Table 2:** Plasmids used in this study

Plasmid	Characteristics	Reference
pK19-*mobsacB*	Contains negative (*sacB*) and positive (Kan^r^) selection markers for genomic integration and deletion, MCS cut with EcoRI/BamHI	Schäfer et al. [34]
pK19-*mobsacB*-P_*hrtB*_-*pfkA*	Plasmid for integration of the *hrtB-*sensor construct upstream of *pfkA*	This work
pK19-*mobsacB*-P_*hrtB*_-*aceE*	Plasmid for integration of the *hrtB-*sensor construct upstream of *aceE*	This work
pK19-*mobsacB*-P_*hrtB*_-*pgi*	Plasmid for integration of the *hrtB-*sensor construct upstream of *pgi*	This work
pK19-*mobsacB*-P_*hrtB*_-*hisD*	Plasmid for integration of the *hrtB-*sensor construct upstream of *hisD*	This work
pK19-*mobsacB*-Δ*cydD*	Plasmid for deletion of *cydD*	This work
pJC1-P_*hrtB*_-*eyfp*	Derivative of pJC1-venus-term-BS, containing *eyfp* under the control of the promoter P_*hrtB*_	Heyer et al. [20]

### Selection on agar plates

Pre-cultures of *C. glutamicum*::P_*hrtB*_-*pfkA* and::P_*hrtB*_-*aceE* each containing pJC1*-*P_*hrtB*_*-eyfp* were inoculated in 5 mL BHI supplemented with 25 µg/mL kanamycin and incubated over night at 30 °C, 170 rpm. On the following day, pre-cultures were diluted in 0.9% NaCl and the dilutions 10^− 3^ to 10^− 5^ were streaked out on CGXII agar plates supplemented with 2% glucose, 100 µM FeSO_4_ (iron excess) and kanamycin. Plates were incubated at 30 °C and pictures taken every 24 h. Clones were picked, covering diverse colony morphologies and fluorescence intensities as observed with a stereomicroscope (Nikon SMZ18 (λ_Ex_: 500/20, λ_Em_: 535/30)).

### Automated f(luorescent)ALE for selection in liquid media

In triplicates, pre-cultures of *C. glutamicum*::P_*hrtB*_-*pfkA* containing pJC1*-*P_*hrtB*_*-eyfp* were inoculated in 1 mL complex medium BHI, and 20 µL were used to inoculate the first main culture well in the microbioreactor. The medium for main cultures in the microbioreactor ALE was CGXII containing 2% glucose, 100 µM FeSO_4_ (i.e. iron excess) and kanamycin. Biomass and fluorescence of active cultures were monitored online, and employed to trigger automated inoculation of subsequent batches. The inoculation of all subsequent batches was triggered when > 50% of the currently active cultures reached a backscatter threshold of 120. New batches were prepared with 770 µL fresh medium, and 30 µL inoculum drawn from the culture with highest specific fluorescence. Cultures with lower specific fluorescence were not used for inoculation. Backscatter and fluorescence signals were smoothed with a crossvalidated smoothing spline [37] to improve signal stability. Completed batches were harvested to a cooled storage position. The three final batches were streaked on BHI plates for single clone selection.

### Whole genome sequencing

Whole genome sequencing (WGS) of isolated *C. glutamicum* clones was performed using next generation sequencing (NGS). First, genomic DNA was prepared using NucleoSpin microbial DNA kit (Macherey-Nagel, Düren, Germany) according to manufacturer’s instructions. Concentrations of the purified genomic DNA were measured using Qubit 2.0 fluorometer (Invitrogen, Carlsbad, CA, United States) according to manufacturer’s instructions. The purified genomic DNA was used for the preparation for genome sequencing using NEBNext Ultra II DNA Library Kit for Illumina (New England BioLabs, Frankfurt am Main) and MiSeq Reagent Kit v2 (300-cycles) (Illumina, San Diego, CA, United States), according to manufacturer’s instructions. A MiSeq system (Illumina, San Diego, CA, United States) was used for sequencing. Data analysis and base calling were accomplished with the Illumina instrument software. FASTQ output files were analyzed for single nucleotide polymorphisms using BV-BRC web resources (3.34.11, https://www.bv-brc.org/) [38] via variation analysis and CLC Workbench 20.0.4 (https://digitalinsights.qiagen.com/) for structural variants and InDel analysis.

### Heme measurements

Measurement of heme contents in the cells was performed using the Hemin Assay Kit (Sigma-Aldrich, St. Louis, United States), with the following preparations and adjustments: A pre-culture was inoculated in 1 mL BHI from a fresh agar plate from either WT or Δ*cydD* in triplicates and incubated at 30 °C for ~ 8 h. Then, 100 µL of pre-culture 1 were transferred to 900 µL CGXII supplemented with 2% glucose and 100 µM FeSO_4_ and incubated at 30 °C overnight (~ 16 h). A respective amount of these cultures according to an OD_600_ of ~ 1 was harvested and further diluted 1:5. 150 µL of this samples was mixed with the provided hemin buffer, vortexed and transferred to a glassbead tube for cell disruption using Precellys^®^ homogenisator (VWR International, PA, United States). 50 µL was taken once as sample, once as blank. Further, the kit was used according to manufacturer’s instructions. Prepared samples and blanks were transferred to a microtiter plate and recorded in a Tecan Microplate Reader (Tecan Trading AG, Switzerland) (absorbance measurement 570 nm, kinetic mode) after 30 min.

## Results

### Design of growth-coupled heme-responsive strains

**Fig. 1:**
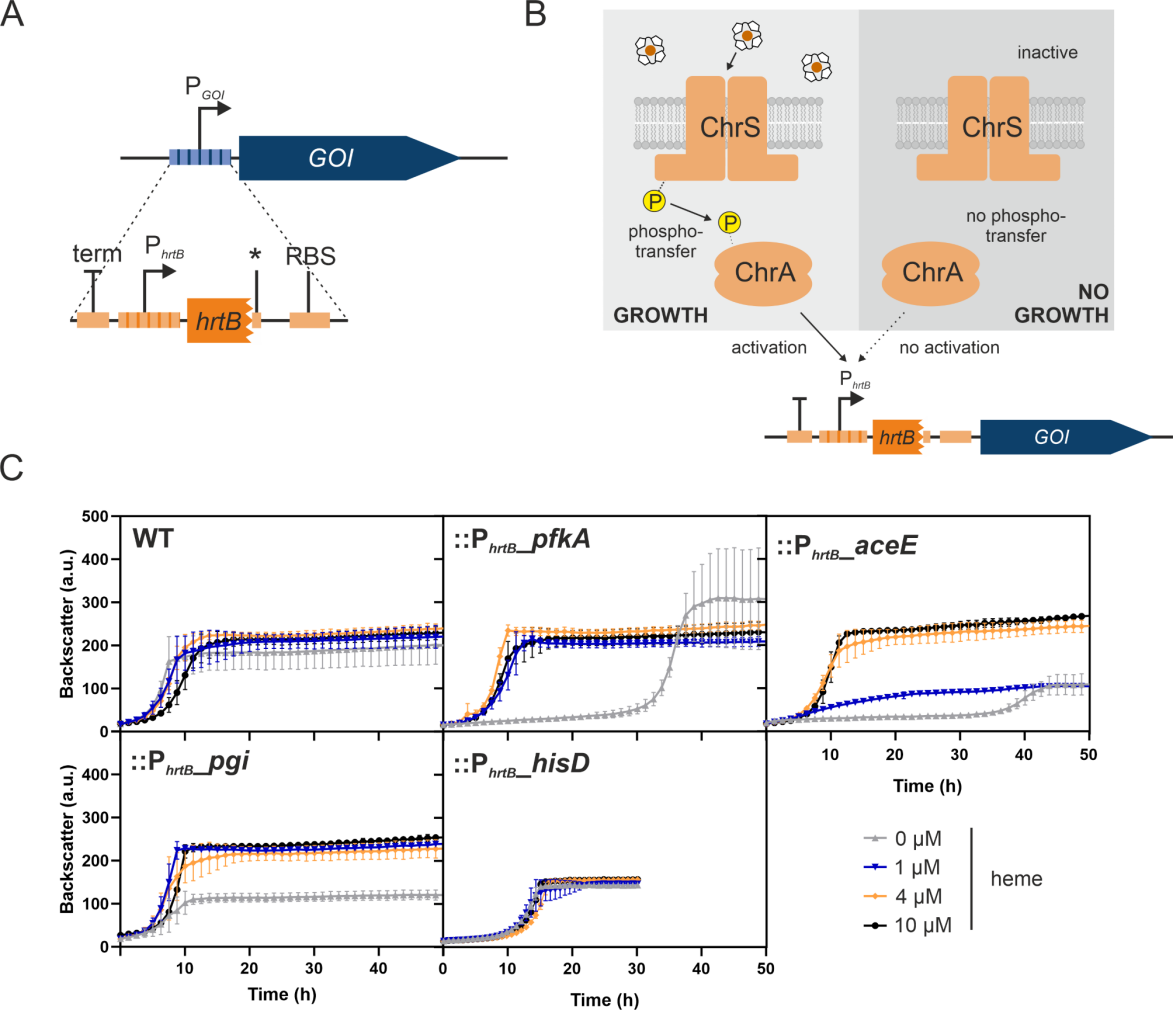
Characteristics of growth-coupled heme biosensor strains *C. glutamicum*::P_*hrtB*_-*pfkA*,::P_*hrtB*_-*aceE*,::P_*hrtB*_-*pgi* and::P_*hrtB*_-*hisD*. (**A**) Schematic overview of the growth-coupled heme biosensor. The integration cassette (orange) consists of a terminator (term) to block remaining promoter activities of upstream regions, the promoter region of *hrtB* (P_*hrtB*_) including the first 30 bp of *hrtB*, followed by stop codon (*), a ribosomal binding site (RBS) and a linker. This cassette was used to exchange the original promoter region of the growth-regulating gene of interest (GOI), i.e. *pfkA*,* aceE*,* pgi* or *hisD*. (**B**) Schematic and simplified overview of::P_*hrtB*_-GOI biosensor activation. When the histidine kinase ChrS is activated by the presence of heme it undergoes autophosphorylation with consequent phosphotransfer to the response regulator ChrA. ChrA gets active as transcriptional regulator, activating the expression of *hrtB*, allowing expression of the GOI and subsequent growth. P = phosphate. (**C**) The strains *C. glutamicum* WT,::P_*hrtB*_-*pfkA*,::P_*hrtB*_-*aceE*,::P_*hrtB*_-*pgi* and::P_*hrtB*_-*hisD* were inoculated to an OD_600_ of 1 in CGXII media containing 2% glucose and 36 µM FeSO_4_ and 0, 1, 4 or 10 µM heme. Growth curves are based on the backscatter measurements in a microbioreactor cultivation system. *n* = 3 biological replicates

A prerequisite for facilitating selection of clones featuring enhanced *hrtB* expression is the coupling of elevated expression levels to cellular growth. To impose such kind of selection pressure, a transcription factor based biosensor construct was generated (Fig. 1A). This construct made use of the heme-responsive two-component system ChrSA, where ChrA activates the expression of the *hrtBA* operon in response to elevated heme levels. Heme is sensed by the sensor kinase ChrS, likely via an intramembrane sensing mechanism [39]. In the sensor strains, the P_*hrtB*_ promoter region was placed in front of different growth-regulating genes of interest, rendering their respective expression and consequently cellular growth dependent on *hrtB* expression and potentially on increasing heme levels. For this purpose, four different genes of interest were tested, based on the studies by Stella et al. [27]: (i) *pfkA* encoding the phosphofructokinase involved in central carbon metabolism, (ii) *aceE* coding for the E1 subunit of the pyruvate dehydrogenase complex linking glycolysis and TCA, (iii) *pgi* encoding the glucose-6-phosphate isomerase catalyzing the reversible reaction from glucose-6-phosphate to fructose-6-phosphate and (iv) *hisD* coding for the histidine-dehydrogenase involved in L-histidine synthesis. Resulting biosensor constructs::P_*hrtB*_-*pfkA*,::P_*hrtB*_-*aceE*,::P_*hrtB*_-*pgi* and::P_*hrtB*_-*hisD* were tested for their growth inhibition in the absence of heme and their consequent suitability for sensor-based selection or ALE approaches. Sensor strains as well as the WT control were cultivated with different heme concentrations (Fig. 1B) and showed varying degrees of growth deficiency. In particular *C. glutamicum*::P_*hrtB*_-*pfkA* and::P_*hrtB*_-*aceE* strains showed a significantly extended lag phase (~ 30–35 h) in the absence of heme. While *C. glutamicum*::P_*hrtB*_-*pfkA* still reached WT-level regarding final biomass,::P_*hrtB*_-*aceE* showed significantly reduced backscatter values. In the case of *C. glutamicum*::P_*hrtB*_-*pfkA*, addition of 1 µM heme already counteracted this growth defect completely, while::P_*hrtB*_-*aceE* restored full growth only upon addition of 4 µM heme. Consequently, the latter construct was less sensitive thereby probably imposing a higher selection pressure. In contrast to the *pfkA* and *aceE*-based strain designs, *C. glutamicum*::P_*hrtB*_*-pgi* and::P_*hrtB*_-*hisD* still showed comparably good growth in the absence of heme and were therefore found to be not suitable for following plate-based selections and ALE experiments.

### Sensor strains::P_*hrtB*_-*pfkA* and::P_*hrtB*_-*aceE* facilitate selection on solid media

Having successfully demonstrated evolutionary pressure towards enhanced *hrtB* expression, we evaluated the suitability of the growth-coupled strains::P_*hrtB*_*-pfkA* and::P_*hrtB*_*-aceE* to conduct selection of strains on solid media. To visualize *hrtB* expression via a fluorescent signal, the parental strains were additionally transformed with the reporter plasmid pJC1-P_*hrtB*_*-eyfp* [20].

**Fig. 2:**
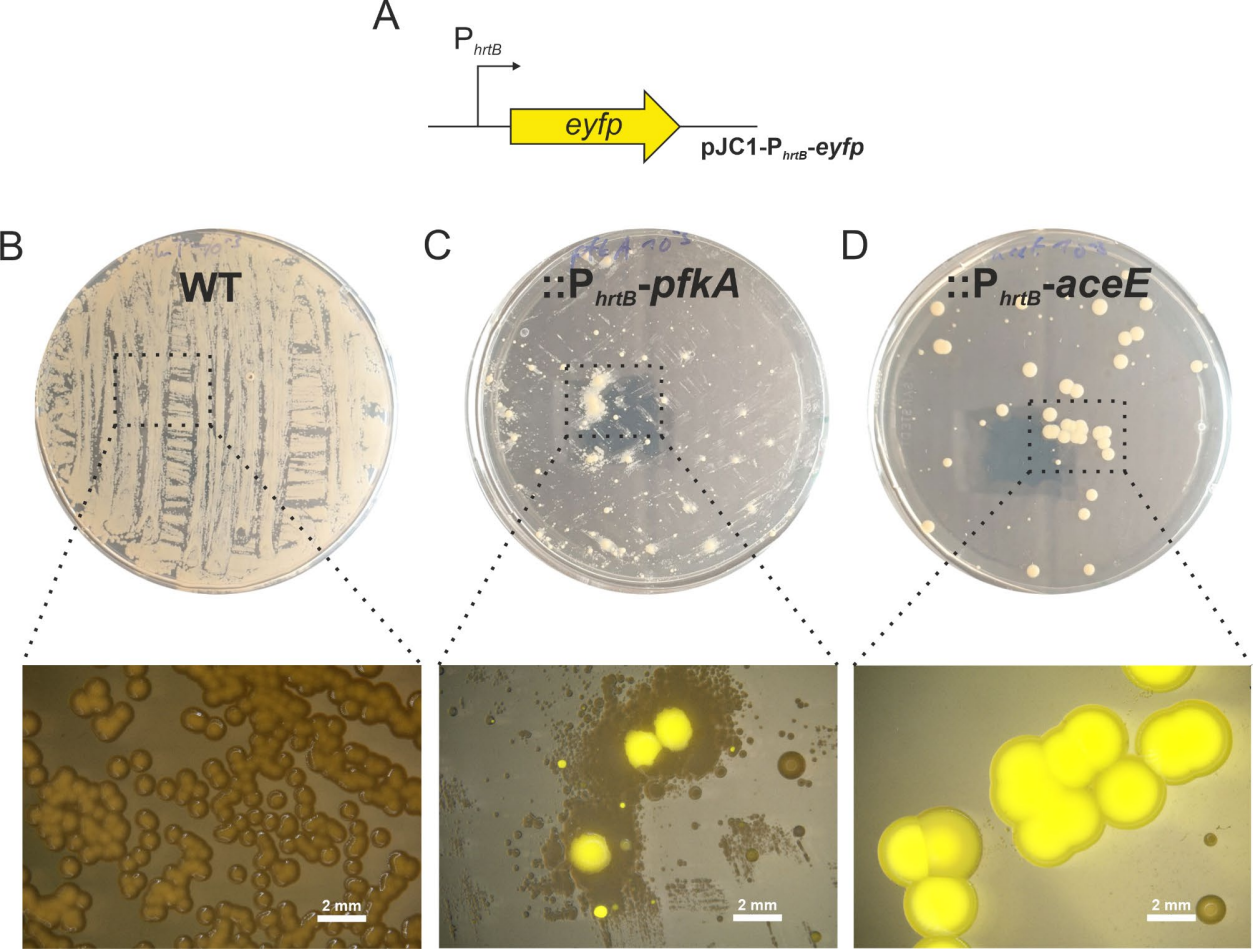
Selection on plates using growth-coupled biosensor strains. Growth and fluorescence of different growth-coupled biosensor strains of *C. glutamicum* ATCC 13032 transformed with (**A**) the reporter plasmid pJC1-P_*hrtB*_-*eyfp*, coupling fluorescence to *hrtB* reporter output. These different strains encompass (**B**) WT, (**C**)::P_*hrtB*__*pfkA* and (**D**)::P_*hrtB*__*aceE* grown on CGXII medium supplemented with 2% glucose, 100 µM FeSO_4_, and 25 µg/mL kanamycin on a 15 g/L agar-agar plate. Photographs of agar plates and single colonies were taken with the stereomicroscope after 96 h of incubation. Close-up images were captured after 96 h using a stereomicroscope Nikon SMZ18 (λ_Ex_: 500/20, λ_Em_: 535/30)

Based on the findings by Stella et al. [27], selection on agar plates was tested as a first approach, since this strategy was found to prevent the enrichment of cheater strains, which was observed in repetitive-batch liquid cultivations. Hereby, cheater strains are defined as those that escape the generated selection pressure by mutating the inserted biosensor region, i.e. regarding this setup, cheaters would not feature increased *hrtB* expression, but would still manage to restore growth. After inoculation of a liquid pre-culture in complex medium BHI, growth-coupled biosensor strains as well as the WT containing the P_*hrtB*_-reporter plasmid were streaked on CGXII agar plates containing 100 µM FeSO_4_, (iron excess conditions) to support heme production. After 72–96 h of inoculation, the growth deficit was also observed on plates (Figure S1-2). Colonies were observed under a stereomicroscope for fluorescent signals indicative of enhanced P_*hrtB*_-reporter output (Fig. 2, Figure S3-4). While the WT control displayed background fluorescence, several large colonies of the biosensor strains::P_*hrtB*_*-pfkA* and::P_*hrtB*_*-aceE* exhibited increased eYFP fluorescence. Interestingly, the colonies exhibited different sizes indicating the diversity of genetic modifications occurred in the selected clones. However, there was no general trend observable correlating eYFP fluorescence of the P_*hrtB*_*-*reporter to the colony size (Figure S5). Interestingly, we observed the growth of non-fluorescent small colonies surrounding big and highly fluorescent colonies of the sensor strain *C. glutamicum*::P_*hrtB*_*-pfkA*, probably benefiting from the secretion of an intermediate glycolytic compound.

For both constructs, > 25 colonies of different sizes and fluorescent intensities were picked for further analysis. Bigger colonies which did not show a fluorescent signal were classified as cheater strains and therefore not selected.

### Automated fluorescent ALE (fALE)

**Fig. 3:**
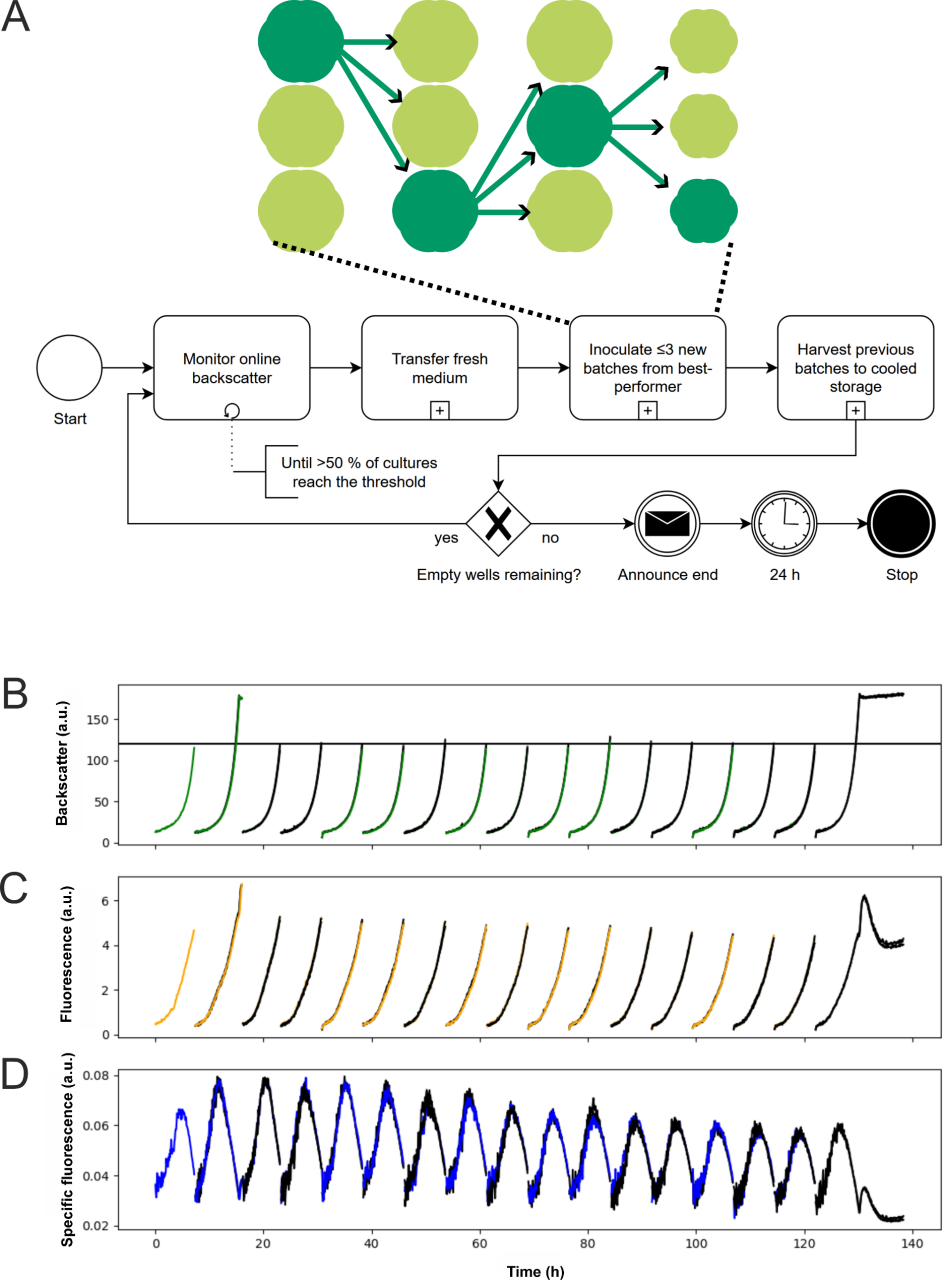
Automated fluorescence ALE of *C. glutamicum* biosensor strains in repetitive batch cultures. (**A**) Depiction of fALE experimental workflow. The automated fALE workflow is depicted in BPMN 2.0 notation (bottom) showing the control flow of the experiment. After the start, backscatter of active cultures was continuously monitored until the predefined threshold was reached by at least half of the active cultures. Once the threshold was reached, fresh medium was transferred to up to 3 FlowerPlate wells and inoculated from the culture with best-performing specific fluorescence (top). Previous cultures were harvested to a cooled storage to facilitate subsequent analysis. After inoculation of a fresh batch, the loop continued with culture monitoring, unless no unused, empty wells remain. Once empty wells remained, a message was sent to the operator to announce the workflow termination 24 h later. Monitored (**B**) backscatter, (**C**) fluorescence and (**D**) specific fluorescence (a.u.) of the fALE workflow of *C. glutamicum* ATCC 13032::P_*hrtB*_-*pfkA*, transformed with a pJC1-P_*hrtB*_-*eyfp* reporter plasmid in CGXII medium supplemented with 2% glucose, 100 µM FeSO_4_, and 25 µg/mL kanamycin, are summarized

As depicted by [27], repetitive batch ALE experiments in liquid cultures of growth-coupled biosensors provoke the enrichment of cheater strains. However, these previous approaches were based on transfers of stationary cultures (after 48 h) into fresh medium and did not take additional parameters, such as fluorescence into account. In this study a repetitive batch ALE cultivation using a robotics platform facilitating automated re-inoculations based on fluorescence was performed. To this means, the *C. glutamicum*::P_*hrtB*_*-pfkA* strain containing the P_*hrtB*_-reporter plasmid was inoculated in CGXII containing 2% glucose, 100 µM FeSO_4_ and kanamycin. The automated workflow performed 17 consecutive batches, out of which all except the first and the last were triplicates (Fig. 3). A programming error led to the 2nd batch exceeding the threshold until the experiment continued as intended.

Samples of the last three batches were streaked on agar plates to enable isolation of single clones. Three clones from each of these three populations underwent Sanger sequencing tests to exclude cheater mutations in the biosensor region. Notably, from in total nine analyzed clones only one represented a classical cheater strain.

### Analysis of selected::P_*hrtB*_-*pfkA* and::P_*hrtB*_-*aceE* clones

**Fig. 4:**
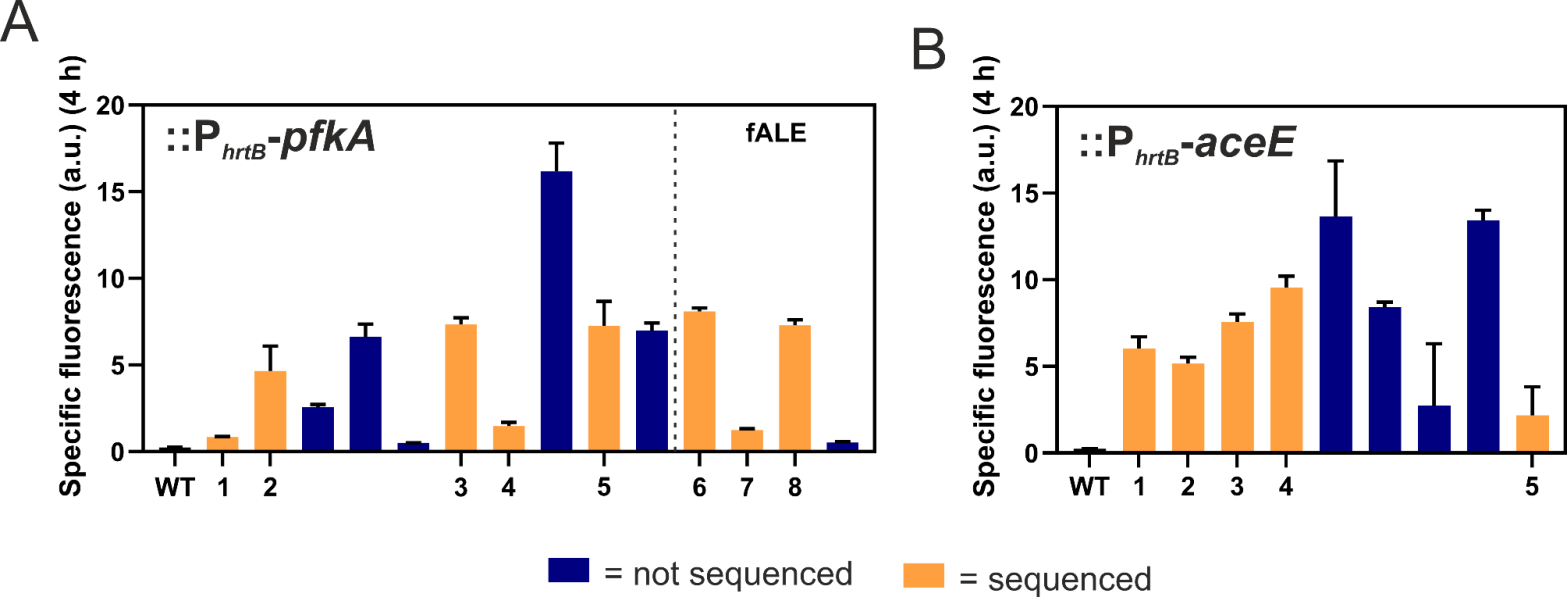
Screening of selected::*P*_*hrtB*_*-pfkA* and::*P*_*hrtB*_*-aceE* clones for increased *hrtB* expression. After a pre-screening in unicates (Figure S3 and S4), several clones of *C. glutamicum* (**A**)::P_*hrtB*_-*pfkA* and (**B**)::P_*hrtB*_-*aceE* were selected and inoculated together with the WT in triplicates to an OD_600_ of 1 in CGXII media containing 2% glucose and 100 µM FeSO_4_. Orange bars represent clones selected for whole genome sequencing. Specific fluorescence at a time point of 4 h (highest *hrtB* reporter output [20]) is given by measured fluorescence (a.u.) divided by respective backscatter (a.u.) measured in a microbioreactor system. *n* = 3 biological replicates. fALE = clones of the fluorescent ALE

In total, 19 clones were selected from plate screening and 4 clones from repetitive batch ALE for further analysis. Specific fluorescence of clones was quantified as a read-out for *hrtBA* expression in a microtiter cultivation system using the P_*hrtB*_-reporter plasmid pJC1-P_*hrtB*_-*eyfp*. Since maximal reporter output was observed at 4 h (Figure S6), this time point was compared for all clones analyzed [20]. A full pre-screening in unicates is depicted in Figure S3-4, while Fig. 4 summarizes reporter output results in triplicates of clones that were chosen for whole genome sequencing (WGS) (Fig. 4 and Figure S6). Strains exhibiting varying reporter outputs were deliberately selected to increase the likelihood of identifying a range of causal mutations through genome sequencing. Note that strains with an extremely high reporter output were not selected in order to avoid to select exclusively for previously reported *chrS* mutations, but rather to cover a variety of causal mutations [23]. Besides reporter assays, qPCR experiments further confirmed the elevated *hrtB* expression of selected clones in comparison to the WT (Figure S7). The expression levels were varying, while not being as extremely high as for a plasmid-based overexpression via pEKEx2-lacI-P_*tac*_-*hrtBA* [40], which faces growth defects [23].

### Identification of beneficial mutations promoting *hrtB* expression

WGS of the selected clones led to the revelation of several mutations, with the key mutations depicted in Table 3 and further mutations summarized in Table S2. From the identified mutations, we can categorize overall four different classes of mutations established with this growth-coupled biosensor approach: (i) mutations in *chrS*, (ii) *chrA* (iii) a mutation in *cydD* as well as (iv) cheater strains, with mutations in the promoter region. Out of seven mutations found in the *chrS* gene, none of them affected the transmembrane domain, which is required for heme sensing. Three of them affected the dimerization and histidine phosphotransfer (DHp) domain (*pfkA*3, *aceE*3 and *pfkA*4), while the other four were located in the catalytic and ATPase (CA) domain (*pfkA*2, *aceE*1, *aceE*2 and *aceE4*). Note that the four remaining clones of the fALE were excluded from the WGS, since previous Sanger sequencing already revealed mutations within the *chrS* region. Regarding the *chrA* mutations, all four mutations were located in the sequence encoding the receiver (REC) domain that contains the conserved aspartate for phosphotransfer, while no mutation was found in the output domain, which is required for DNA-binding. Interestingly, *chrA* mutations were mainly found via the repetitive batch ALE and only one was obtained from plate screenings of::P_*hrtB*_-*aceE*. Last but not least, selection on plates using::P_*hrtB*_-*pfkA* strain resulted in the isolation of a clone featuring a mutation in *cydD* located in the annotated nucleotide binding domain. While mutations in *chrS* were previously reported to enhance *hrtB* expression and confirmed the functionality of the sensor-based selection approach [23], the mutation in *cydD* encoding subunit II of an ABC transporter essential for cytochrome *bd* oxidase assembly was of particular interest and further characterized in the following.

**Table 3:** Potential key mutations identified in the sensor strains *C. glutamicum*::P_*hrtB*_-*pfkA* and::P_*hrtB*_-*aceE*

Clone	Mutation	Gene	Annotation
*pfkA*1	Exchange V469G	*cydD* (cg1299)	ABC transporter, subunit II, essential for cytochrome *bd* oxidase assembly
*pfkA*1	Exchange L460F		
*pfkA*2	Deletion L348fs	*chrS* (NCgl1935)	two component sensor kinase, control of heme homeostasis/export
*pfkA3*,* aceE3*	Exchange A190V		
*pfkA4*	Deletion K205fs		
*aceE1*	Exchange I301M		
*aceE2*	Exchange A328fs		
*aceE4*	Exchange Q313*		
*pfkA5* ^*1*^	Insertion CT		
*pfkA*6, *pfkA*8	Exchange A104V	*chrA* (NCgl1934)	Two-component transcriptional response regulator, LuxR family
*pfkA*7, *aceE*5	Exchange A124V		

^1^In this clone, the mutation occurred in the intergenic/promoter region of *chrS* in a fraction of 40%, holds potential to be a cheater strain

### Deletion of *cydD* leads to enhanced cellular heme levels

**Fig. 5:**
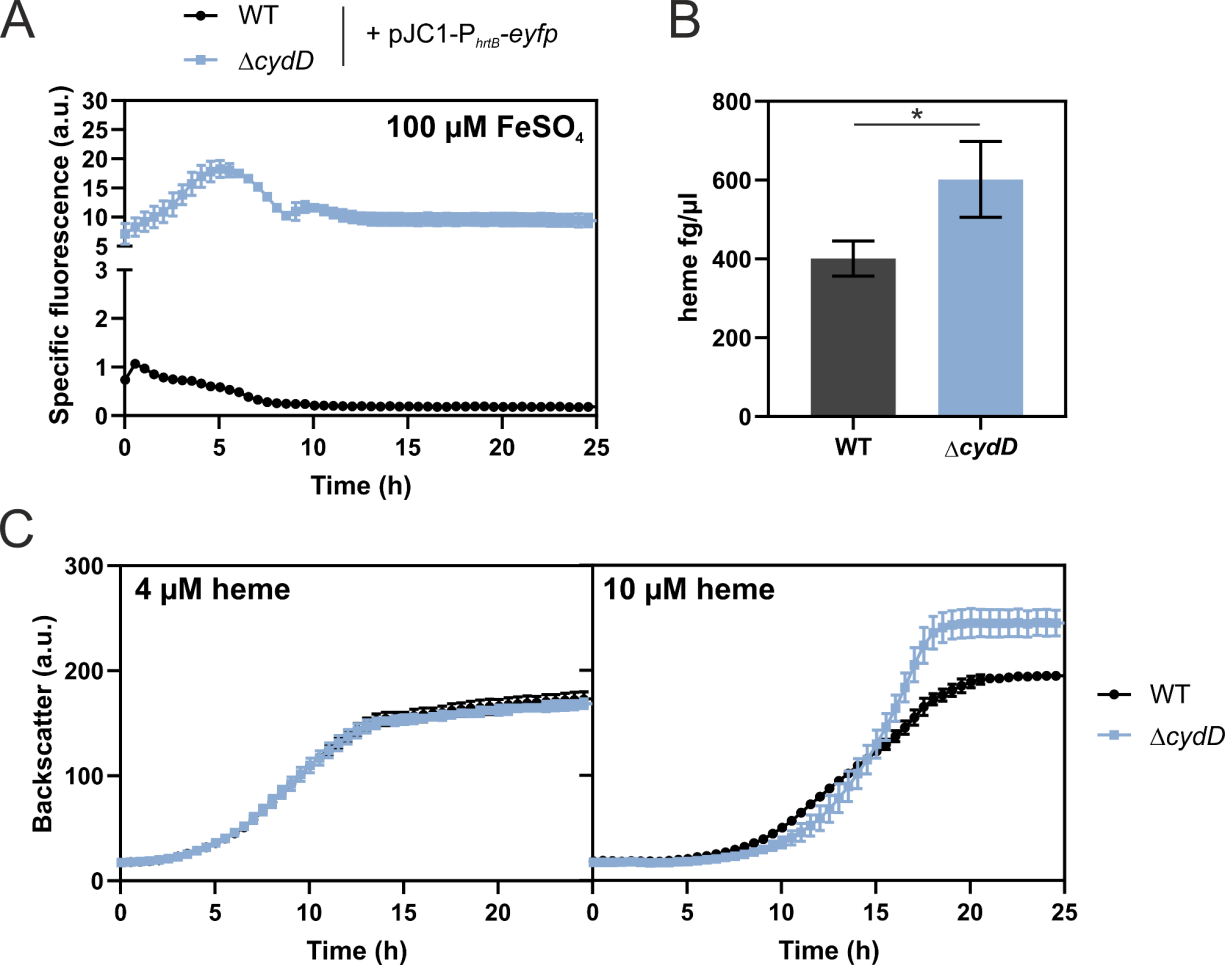
Analysis of a *cydD*-deficient *C. glutamicum* strain. (**A**) The *C. glutamicum* WT (black) as well as the *cydD* deletion strain Δ*cydD* (blue) were transformed with the plasmid pJC1-P_*hrtB*_-*eyfp*. These were inoculated to an OD_600_ of 1 in CGXII media containing 2% glucose and 100 µM FeSO_4_. Reporter assays visualizing *hrtB*-expression were performed in a microbioreactor cultivation system. Specific fluorescence is given by measured fluorescence (a.u.) divided by respective backscatter (a.u.), with the latter depicted in Figure S9. *n* = 3 biological replicates. (**B**) Total heme content measurement in WT (grey) and Δ*cydD* (blue), from an overnight cultivation in the presence of 100 µM FeSO_4_. *n* = 3 biological replicates. Statistical significance was confirmed by Student’s t-test (P value ≤ 0.05). (**C**) The WT (black) and the *cydD* deletion strain Δ*cydD* (blue) were inoculated to an OD_600_ of 1 in CGXII media containing 2% glucose, no FeSO_4_, but either 4 or 10 µM heme in a microbioreactor cultivation system. *n* = 3 biological replicates

A recent structural analysis of CydDC presents comprehensive evidence that CydDC acts as a heme exporter necessary for proper maturation of cytochrome *bd* in *E. coli* [28]. In our study, two single nucleotide polymorphisms (SNPs) were found in the *cydD* gene of *C. glutamicum*. However reverse engineering of single SNPs did not lead to any significant impact on *hrtB* expression (Figure S8). Therefore, a strain lacking the whole *cydD* gene was constructed and further analysed. Confirming the screening results, deletion of *cydD* resulted in significantly enhanced P_*hrtB*_-reporter output (Fig. 5A), even higher than the originally selected *pfkA*1 clone, with only minor impact on growth (Figure S9). Using a heme assay kit, it was determined that Δ*cydD* showed on top a slightly increased total cellular heme content compared to the WT (Fig. 5B). Enhanced HrtBA activity of the Δ*cydD* strain was furthermore supported by a higher tolerance of this strain to externally added heme when compared to the WT. However, this enhanced tolerance was only observed for concentrations up to 10 µM heme (Fig. 5C), but not as pronounced as for *chrS* mutants [23].

## Discussion

Heme biosynthesis regulation and homeostasis is highly complex and multilayered, as recently reviewed for Gram-positive bacteria [14]. This complexity offers numerous avenues for manipulating heme production within microbial cells. The export of heme via HrtBA in relation to its production is of particular interest. Within the present study, we demonstrated that the usage of growth-coupled biosensors, based on P_*hrtB*_ activation by ChrSA, represent a promising strategy to unravel mutations that potentially improve heme export and enhance cellular heme levels. Furthermore, we emphasize the automated-liquid ALE approach as a promising strategy leading to the isolation of significantly less cheaters than the standard repetitive batch approach [27].

The gene predominantly selected through this plate-based approach and fALE was *chrS*. Previous studies on heme toxicity in *C. glutamicum* reported on different mutations in *chrS* within the CA-domain that affect *hrtB* expression and consequently provide a highly elevated heme tolerance [23]. In these strains, upregulation of *hrtB* expression was no more dependent from ChrS phosphotransfer to ChrA, but from the non-cognate, paralogous histidine kinase HrrS. Due to the catalytic inactivation of ChrS, *hrtB* expression was constitutively high since also the specific phosphatase activity of the cognate kinase was abolished. Although the effect of the resulting pseudokinase variant of ChrS is not conclusively unraveled so far, mutations in *chrS* bear the potential to elevate the heme export and confer an additional advantageous high heme tolerance (> 100 µM of heme).

We further found mutations in *chrA* encoding the response regulator of the ChrSA TCS. The two identified mutations at the alanine positions 104 and 124 are not at positions conserved in the receiver domain of response regulators [41]. These mutations are unlikely to cause a loss of function, as deletion of *chrA* is not resulting in increased P_*hrtB*_-reporter output, but a complete abolishment of transcriptional activation [21]. In literature there are several examples of amino acid substitutions that activate RRs, as summarized by Smith and Latiolais [42]. Such RR activating strategies include resistance to dephosphorylation or enhanced phosphorylation leading to more RR in the activated state. Similar effects can also be hypothesized for the underlying *chrA* mutants. Mutations could in general influence the interaction of the RR with the cognate or non-cognate histidine kinases or reduce the dephosphorylation capacity mediated by ChrS.

Finally, both *chrS* and *chrA* mutations demonstrate a promising route to increase *hrtB* expression for optimal heme production. These targets are of special interest as, to the best of our knowledge, the *hrtBA*-specific TCS ChrSA has not yet been engineered for enhanced heme production in *C. glutamicum*. Recent studies, rather focused on the paralogous TCS HrrSA, which regulates more than 200 targets involved in heme homeostasis [22]. Here, strains were engineered lacking the *hrrS* gene to minimize heme binding in the cell envelope [13]. However, this as well as our previous study [23] emphasize promising targets for heme export engineering resulting in exceptionally high HrtBA activity, wildtypic growth and high heme tolerance. Based on these results, we therefore highlight the very specific and heme-responsive TCS ChrSA as engineering target in *C. glutamicum*.

Beyond classical heme regulatory TCS circuits, the growth-coupled biosensor selection yielded a mutant of *cydD*, which codes for a subunit of the annotated CydDC ABC transporter encoded in the *cydABCD* operon [43]. Several studies described an essential role of CydDC in the assembly and maturation of cytochrome *bd* oxidase [44, 45], as encoded by *cydAB* in *C. glutamicum*. Across several controversies throughout the years concerning the exact role of CydDC, the connection to heme is often discussed, as it is indispensable as cofactor for the cytochrome *bd* oxidase [43, 46, 47]. Interestingly, a recent study provided evidence that CydDC is additionally transporting heme itself in *E. coli*, probably for direct usage as cofactor for the cytochrome *bd* oxidase [28]. If CydD functions as heme exporter for proper cytochrome *bd* oxidase heme cofactor assembly in *C. glutamicum*, deletion of *cydD* might result in the accumulation of heme in the cytoplasmic membrane. Given the hydrophobic nature of heme, accumulation in the cytoplasm appears unlikely. Previous studies already suggested that the ChrS kinase is triggered by an increase in intramembrane heme levels, leading to the activation of *hrtBA* expression via the RR ChrA [39]. This is in contrast to earlier research suggesting that heme is sensed extracellularly [48, 49]. Since heme is a highly hydrophobic molecule, intramembrane signaling appears likely and is in agreement with recent studies.

More than 30 years ago, it was already speculated that *cydD* might have a role in heme biosynthesis in *E. coli* [44, 50]. As we observed elevated heme contents despite increased P_*hrtB*_-reporter output, we hypothesize that CydD(C) has further unknown roles coupled to heme biosynthesis that should be addressed in future studies.

## Conclusion and outlook

Taken together, this study demonstrates the potential of a P_*hrtB*_-based, growth-coupled biosensor ALE approach to uncover so far unknown targets for improving heme production strains that go beyond rational engineering. The presented automated workflow for alternative liquid ALE interfaces with lab automation approaches for strain engineering while resulting in a comparably low isolation frequency of cheater strains. However, a systematic comparison focusing on similar strains and target molecules would be required to provide a comprehensive comparison of the different ALE approaches. In combination with plate-based selections, the biosensor-based approach presented in this study led to the identification of several promising new targets. These targets can be implemented in existing heme production strains to improve heme secretion rates and enhance overall heme productivity.

## Electronic supplementary material

Below is the link to the electronic supplementary material.

Supplementary Material 1

## Data Availability

The datasets generated and/or analyzed during the current study are included in this published article and its supplementary information files. Further data, including raw and cleaned sequencing reads, can be found in the Data Hub https://archive.nfdi4plants.org/records/wr9ps-fmv73. It is available under DOI: https://doi.org/10.60534/wr9ps-fmv73. Further, all sequencing data for this study have been deposited in the European Nucleotide Archive (ENA) at EMBL-EBI under accession number PRJEB79651 (https://www.ebi.ac.uk/ena/browser/view/PRJEB79651).
