# Supplementary material for: Biosensor-based growth-coupling as an evolutionary strategy to improve heme export in Corynebacterium glutamicum
Source: Microb Cell Fact. 2024 Oct 14;23:276. doi: 10.1186/s12934-024-02556-1 (PMC11472508; doi:10.1186/s12934-024-02556-1)
Supplement: Supplementary file 1 — Supplementary Material 1 [file 12934_2024_2556_MOESM1_ESM.pdf]

Supplements to: Biosensor-based growth-coupling as an evolutionary strategy to improve heme export in *Corynebacterium glutamicum*

Aileen Krüger[1], Janik Göddecke[1], Michael Osthege[1], Luis Navratil[1], Ulrike Weber[1], Marco Oldiges[1], Julia Frunzke[1*]

[1] Forschungszentrum Jülich GmbH, Institute for Bio- and Geosciences 1, IBG1, 52425 Jülich, Germany

*** Correspondence:**
Corresponding Author
j.frunzke@fz-juelich.de

Figures:

Figure S1: Growth of different *C. glutamicum* heme-coupled biosensor strains in iron-rich conditions, first round

Figure S2: Growth of different *C. glutamicum* heme-coupled biosensor strains in iron-rich conditions, second round

Figure S3: Screening of evolved ::P*hrtB-pfkA* clones for elevated *hrtB* expression

Figure S4: Screening of evolved ::P*hrtB-aceE* clones for elevated *hrtB* expression

Figure S5: Correlation of *hrtB*-expression at 4 h (peak specific fluorescence) and colony size

Figure S6: Screening of evolved *::P$_{hrtB}$-pfkA* and *::P$_{hrtB}$-aceE* strains for increased *hrtB* expression

Figure S7: qPCR analysis comparing expression levels of *hrtB* in a selection of clones from plate-based selections and fALE in comparison to the *C. glutamicum* WT

Figure S8: Influence on *cydD* single nucleotide polymorphisms on *hrtB* expression

Figure S9: Growth analysis of a *cydD*-deficient *C. glutamicum* strain in iron excess

Tables:

Table S1: Oligonucleotides used in this study

Table S2: Further (non-key) mutations established from ALE experiments with *C. glutamicum* ::P$_{hrtB}$_*pfkA* and ::P$_{hrtB}$_*aceE*

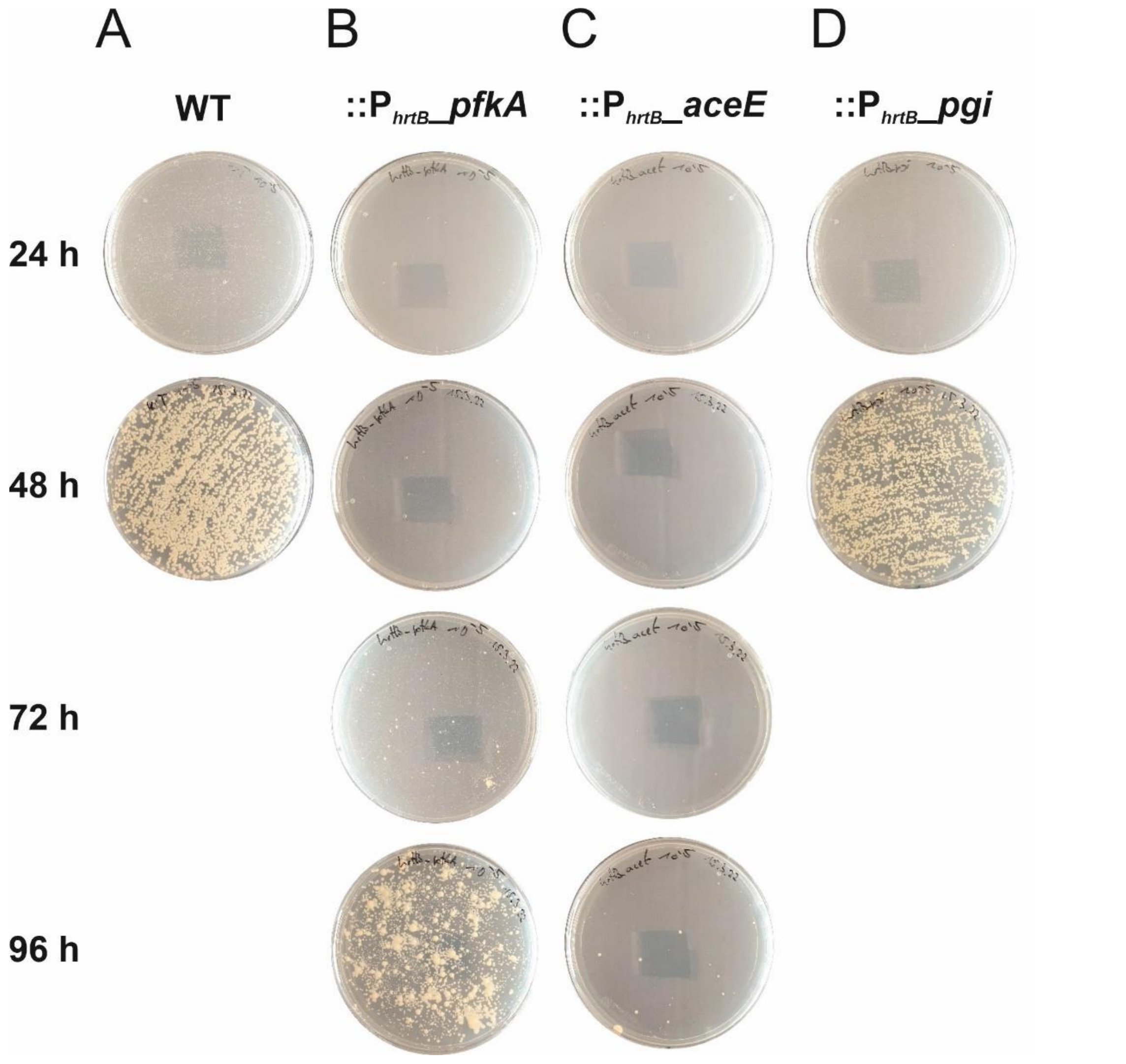

Figure S1: Growth of different *C. glutamicum* heme-coupled biosensor strains in iron-rich conditions, first round. *C. glutamicum* ATCC 13032 (A) WT, (B) ::P_{hrtB}_*pfkA*, (C) ::P_{hrtB}_*aceE* and (D) ::P_{hrtB}_*pgi* were cultivated overnight in BHI, 30°C. Cultures were diluted by a factor of 10^{-5} in 0.9% NaCl and plated on CGXII agar with 2% glucose and an iron excess of 100 µM $FeSO_4$. Photographs were taken after 1, 2, 3 and 4 days of incubation.

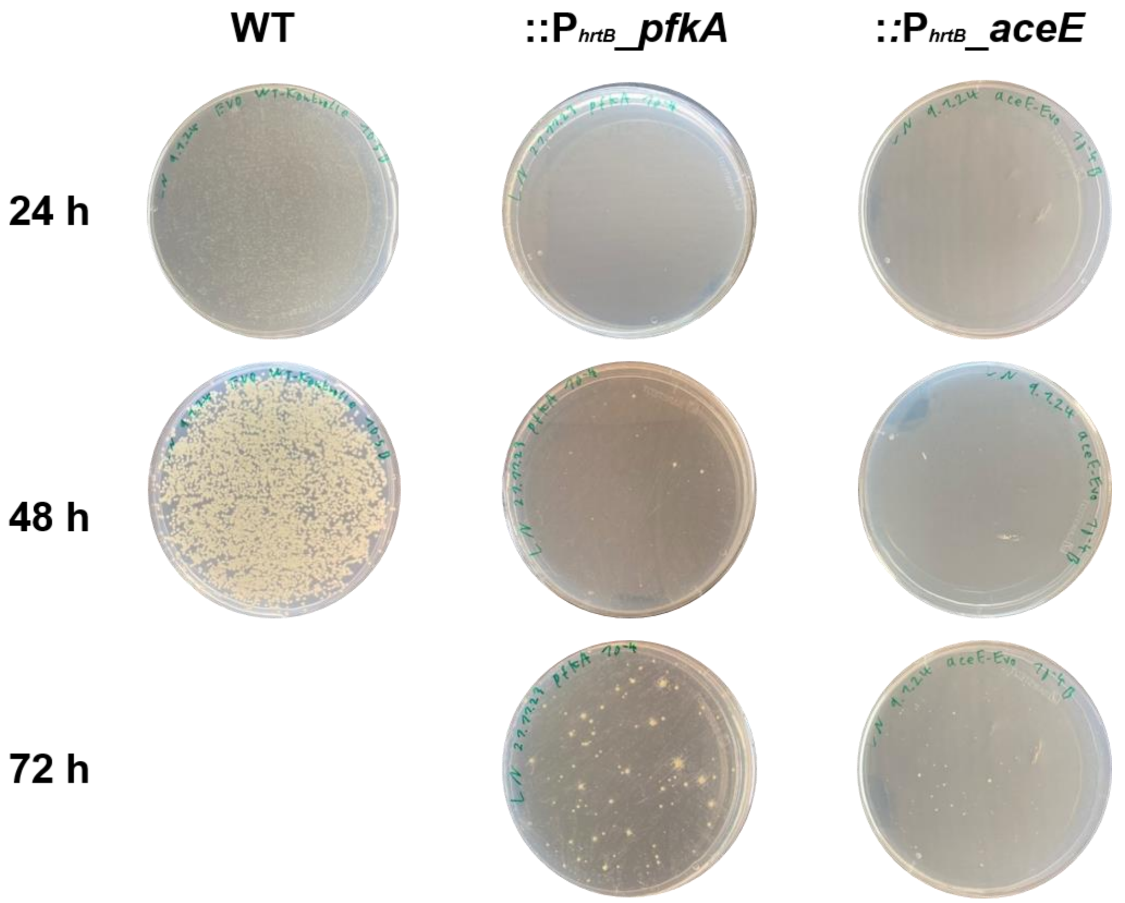

Figure S2: Growth of different *C. glutamicum* heme-coupled biosensor strains in iron-rich conditions, second round. *C. glutamicum* ATCC 13032 WT, ::P$_{hrtB}$_*pfkA* and ::P$_{hrtB}$_*aceE* were cultivated overnight in BHI, 30°C. Cultures were diluted by a factor of 10^{-4} in 0.9% NaCl and plated on CGXII agar with 2% glucose and an iron excess of 100 µM $FeSO_4$. Photographs were taken after 1,2 and 3 days of incubation.

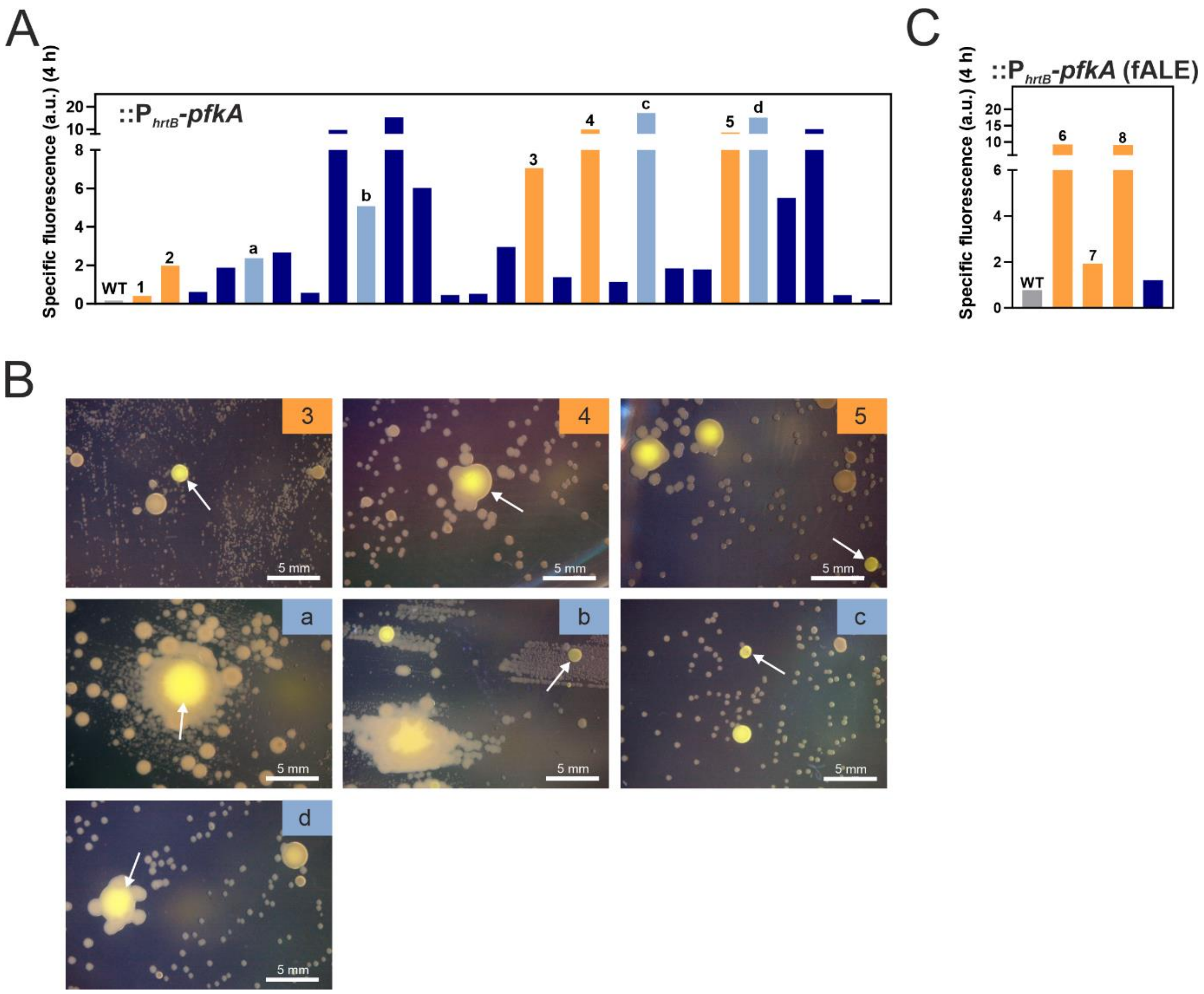

Figure S3: Screening of evolved ::P*hrtB-pfkA* clones for elevated *hrtB* expression. (A) For a screening after adaptive laboratory evolution (ALE) on plates, the *C. glutamicum* WT as well as 27 evolved ::P_{hrtB}-*pfkA* strains containing the plasmid pJC1-P_{hrtB}-*eyfp* were inoculated to an OD_{600} of 1 in CGXII media containing 2% glucose and 100 µM $FeSO_4$. Reporter assays visualizing *hrtB*-expression were performed in a microbioreactor cultivation system. Shown is the specific fluorescence (absolute fluorescence/backscatter) in arbitrary units after 4 h of cultivation. n = 1 biological replicates. Orange bars represent the fluorescent signal of clones that were chosen for further replicate analysis, labelled with their clone number above, as they are also listed within the main text. Light blue bars represent those for which also a microscopy picture is representatively available in (B), but were not chosen for further analysis. (B) Images of the plate evolution of several selected clones of varying sizes and fluorescence intensities were captured using using the stereomicroscope Nikon SMZ18 (λ_{Ex}: 500/20, λ_{Em}: 535/30). Arrows indicate the respective clone. (C) As in (A), four clones of the liquid f(luorescent)ALE are shown, with those chosen for further analysis depicted in orange.

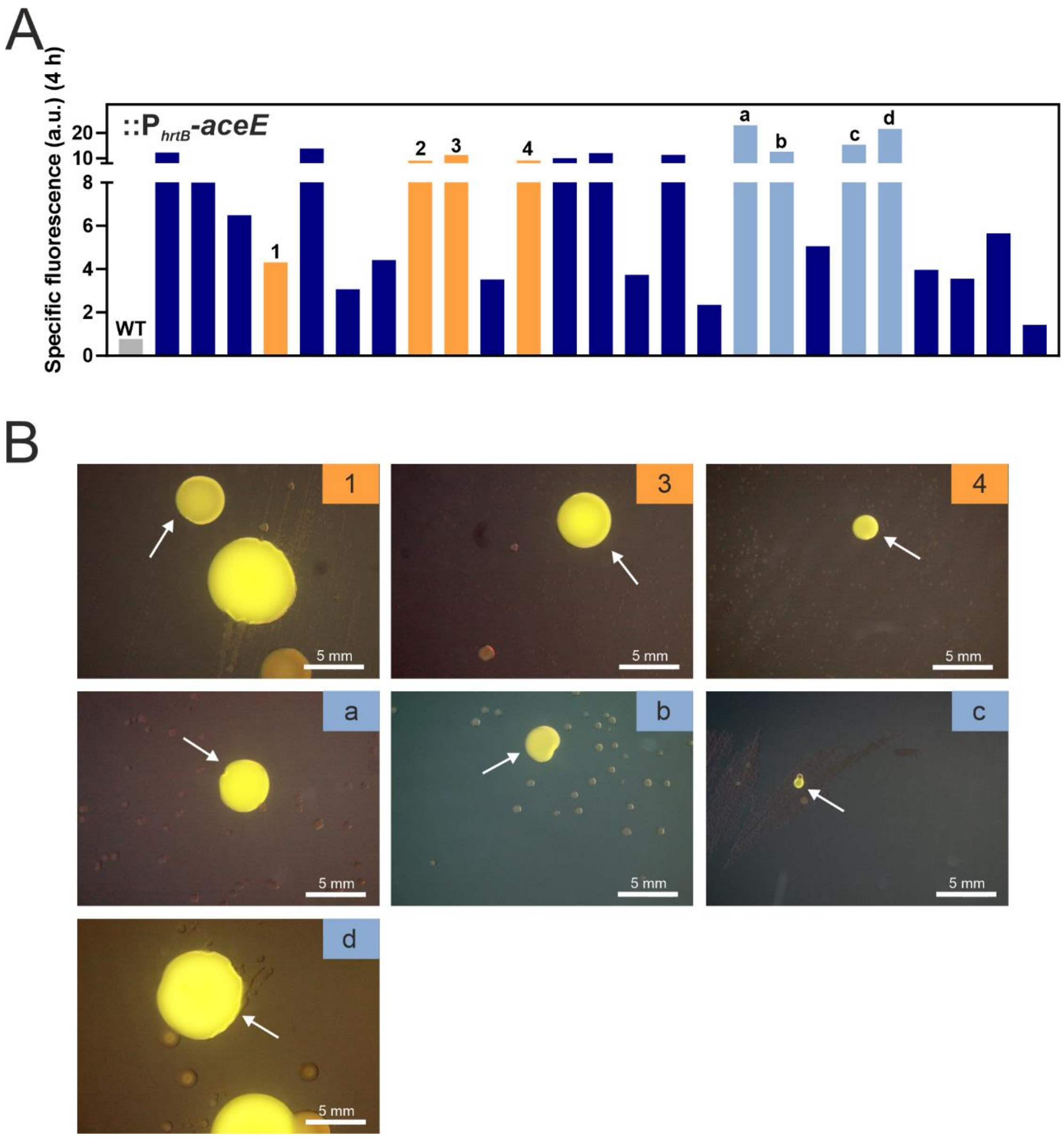

Figure S4: Screening of evolved ::P*hrtB-aceE* clones for elevated *hrtB* expression. (A) For a screening after adaptive laboratory evolution on plates, the *C. glutamicum* WT as well as 27 evolved ::P_{hrtB}-*aceE* strains containing the plasmid pJC1-P_{hrtB}-*eyfp* were inoculated to an OD_{600} of 1 in CGXII media containing 2% glucose and 100 µM $FeSO_4$. Reporter assays visualizing *hrtB*-expression were performed in a microbioreactor cultivation system. Shown is the specific fluorescence (absolute fluorescence/backscatter) in arbitrary units after 4 h of cultivation. n = 1 biological replicates. Orange bars represent the fluorescent signal of clones that were chosen for further analysis, labelled with their clone number above, as they are also listed within the main text. Light blue bars represent those for which also a microscopy picture is representatively available in (B), but were not chosen for further analysis. (B) Images of the plate evolution of several selected clones of differing sizes and fluorescence intensities using stereomicroscope Nikon SMZ18 (λ_{Ex} : 500/20, λ_{Em} : 535/30). Arrows indicate the respective clone.

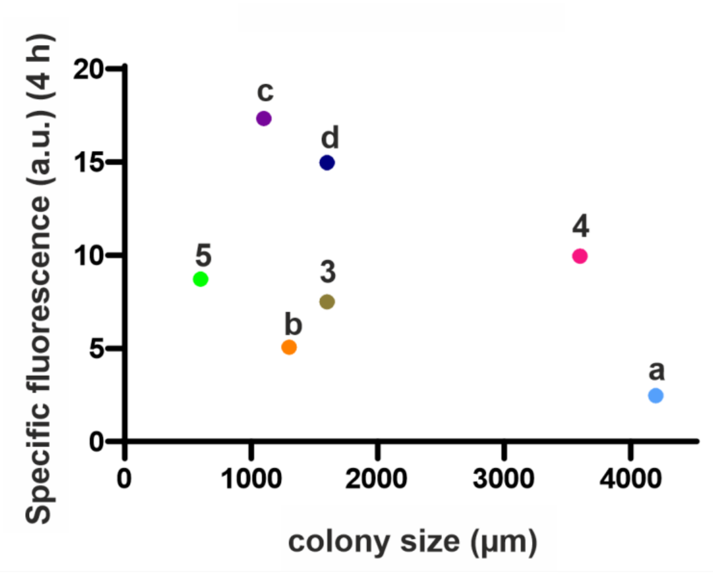

Figure S5: Correlation of *hrtB*-expression at 4 h (peak specific fluorescence) and colony size. The respective clones from the ALE of ::P_{hrtB}-*pfkA* as shown in figure S3 are depicted, labeled each above the value point. Colony size in µm was measured using the measuring tool with the NIS-elements AR 5.30.03 software (Nikon, Japan), while the specific fluorescence was deduced from the microtiter cultivation experiment after 4 h, where specific fluorescence represents the measured fluorescence per backscatter (a.u.).

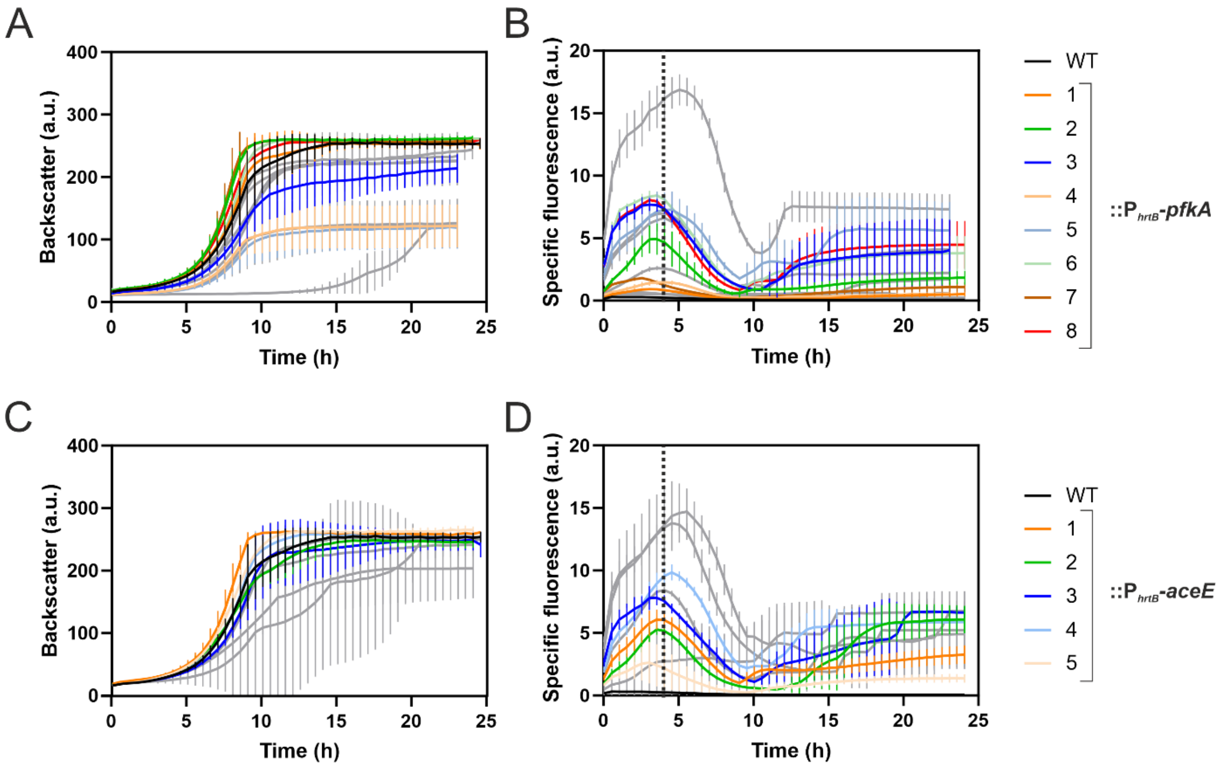

Figure S6: Screening of evolved *::P$_{hrtB}$-pfkA* and *::P$_{hrtB}$-aceE* strains for increased *hrtB* expression. Several evolved clones of *C. glutamicum* (A-B) ::P$_{hrtB}$_*pfkA* and (C-D) ::P$_{hrtB}$_*aceE* were selected and inoculated together with the WT in triplicates to an OD_{600} of 1 in CGXII media containing 2% glucose and 100 µM $FeSO_4$. Backscatter (a.u.) is depicted in (A) and (C), measured in a microbioreactor system. Specific fluorescence as shown in (B) and (D), respectively, is given by measured fluorescence (a.u.) divided by respective backscatter (a.u.). The dotted vertical line represents the timepoint 4 h, as it is summarized in Figure 4 (main text). Clones in grey were not chosen for further analysis. n = 3 biological replicates.

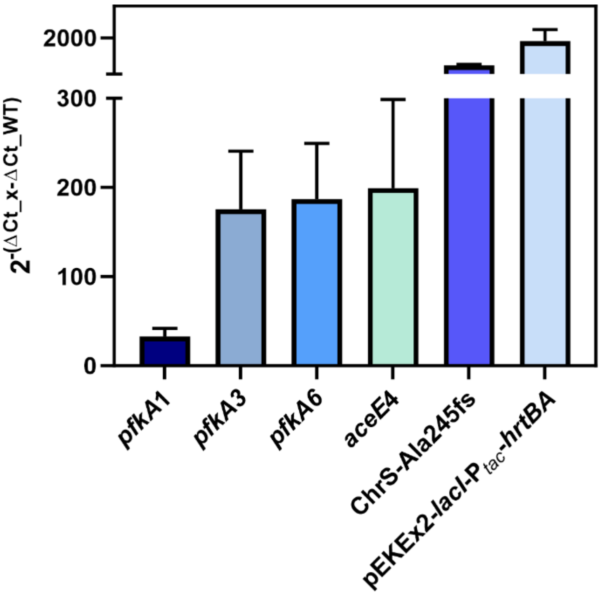

Strain	$2^{-(\Delta Ct\_x-\Delta Ct\_WT)}$
*pfkA*1	32.92 ± 7.34
*pfkA*3	175.43 ± 53.36
*pfkA*6	186.81 ± 51.12
*aceE*4	199.10 ± 81.40
ChrS-Ala245fs	715.17 ± 38.67
pEKEx2-*lacI*-P_{tac}-*hrtBA*	1852.53 ± 436.26

Figure S7: qPCR analysis comparing expression levels of *hrtB* in a selection of clones from plate-based selections and fALE in comparison to the *C. glutamicum* WT. The *C. glutamicum* WT, selected clones *pfkA*1, *pfkA*3, *pfkA*6 and *aceE*4 as well as the pseudokinase variant ChrS-Ala245fs (Krüger and Frunzke, 2022) and a strain harboring the *hrtB*-overexpression plasmid pEKEx2-*lacI*-P_{tac}-*hrtBA* (Heyer et al., 2012) as positive controls were inoculated in 1 mL CGXII medium supplemented with 2% glucose and 100 µM $FeSO_4$ in deep-well plates. After 4 h, the culture was harvested in ice-falcons for RNA isolation via the Monarch Total RNA Miniprep Kit (New England BioLabs, Frankfurt am Main). Using the Luna One-Step RT-qPCR Kit (New England BioLabs, Frankfurt am Main) according to manufacturer's instructions, qPCR was performed in a qTower device (Analytik Jena, Jena). Analysis followed using qPCRsoft 3.1 (Analytik Jena, Jena). The housekeeping gene *ddh* served as reference for normalization. Data represents three biological and two technical replicates. Fold-change was calculated according to the $2^{-\Delta\Delta Ct}$ method (Livak and Schmittgen, 2001).

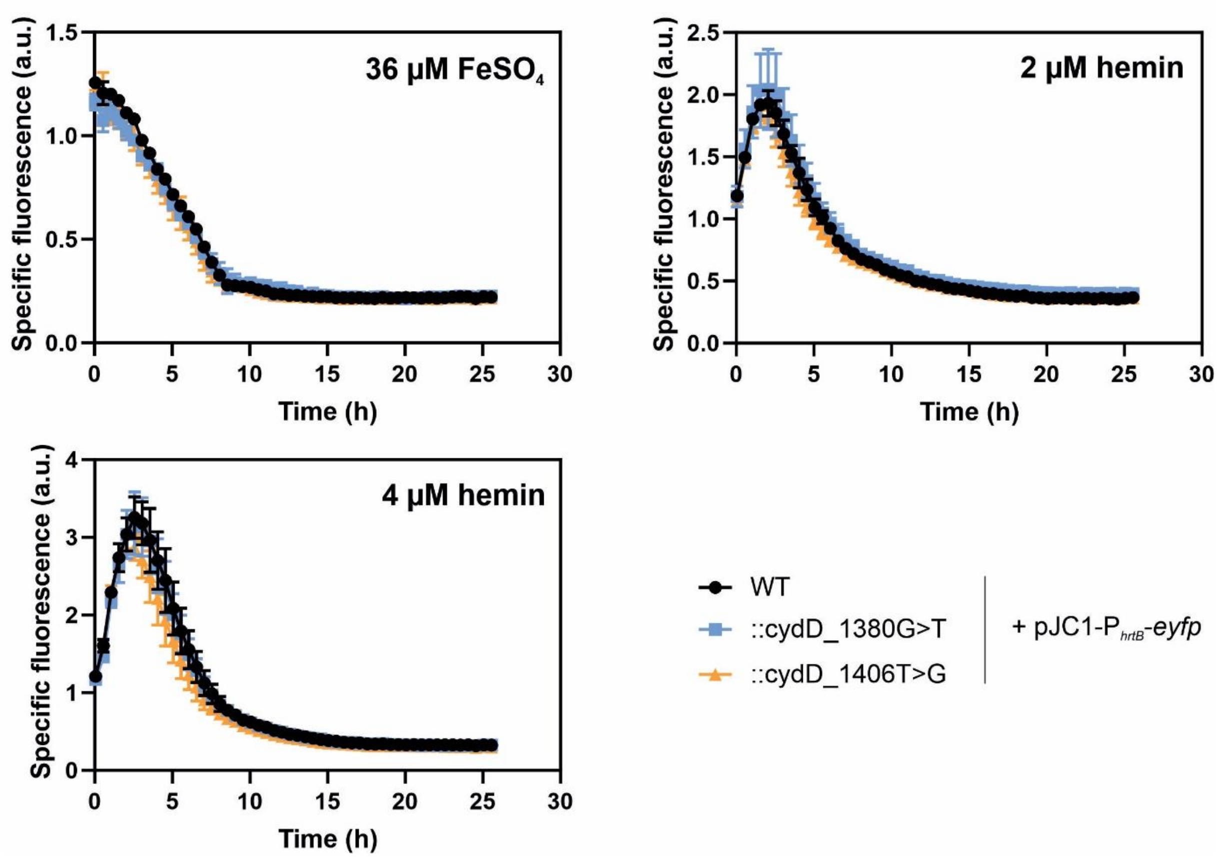

Figure S8: Influence on *cydD* single nucleotide polymorphisms on *hrtB* expression. The mutations 1380G>T and 1406T>G as found in the ALE experiment were separately introduced into *C. glutamicum*. These strains, as well as the WT, were transformed with the reporter plasmid pJC1-P_{hrtB}-*eyfp* (Heyer et al., 2012)and inoculated in triplicates to an OD_{600} of 1 in CGXII media containing 2% glucose and 36 µM $FeSO_4$, or no $FeSO_4$ but 2 µM or 4 µM hemin. Specific fluorescence is given by fluorescence (a.u.) divided by respective backscatter (a.u.) measured in a microbioreactor system. n = 3 biological replicates.

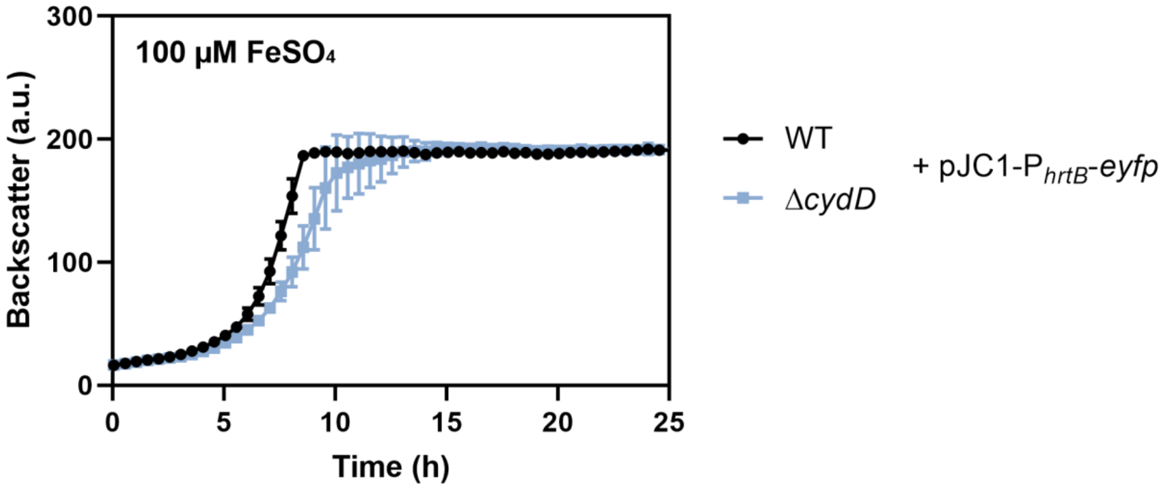

Figure S9: Growth analysis of a *cydD*-deficient *C. glutamicum* strain in iron excess. (A) The *C. glutamicum* WT (black) as well as the *cydD* deletion strain Δ*cydD* (blue) were transformed with the plasmid pJC1-P$_{hrtB}$-*eyfp*. These were inoculated to an OD_{600} of 1 in CGXII media containing 2 % glucose and 100 µM $FeSO_4$. Growth was monitored as backscatter (a.u.) in a microbioreactor system. n = 3 biological replicates. Respective *hrtB*-reporter output is depicted in Figure 4.

Table S1: Oligonucleotides used in this study

Oligonucleotide	Sequence 5' → 3'	Use
A416_term_fw	TTTTGGCGGATGAGAGAAGA	Terminator
A417_term_rv	CAAAAGAGTTTGTAGAAACGCA	
A418_IR_hrtB_fw	GTTTCTACAAACTCTTTTGGCCACCACGATAGATCAACT	Intergenic region of *hrtB* and *chrS*
A419_IR_hrtB_rv	ATGATATCTCCTTCTTAAAGTTCATGCGGTGAGTTCTTTTAGTC	
A414_pfkA_leftflank_fw	**CCTGCAGGTCGACTCTAGAG**AGAGTCGCCCCGATAAGTTT	Left flank for
A415_pfkA_leftflank_rv	TCTTCTCTCATCCGCCAAAATCTGACCATCTTATTTAATCGCCA	BS *pfkA*
A420_pfkA_rightflank_fw	TGAACTTTAAGAAGGAGATATCATATGCGAATTGCTACTCTCACG	Right flank for
A421_pfkA_rightflank_rv	**TTGTAAAACGACGGCCAGTG**ATACCTGCGTGCAGAGCAAT	BS *pfkA*
A422_hisD_leftflank_fw	**CCTGCAGGTCGACTCTAGAG**TCCGGTGTCGCTGAAGTTAA	Left flank for
A423_hisD_leftflank_rv	TCTTCTCTCATCCGCCAAAAACCTATTGTATTCCCCACGTAAC	BS *hisD*
A424_hisD_rightflank_fw	TGAACTTTAAGAAGGAGATATCATATGTTGAATGTCACTGACCTGC	Right flank for
A425_hisD_rightflank_rv	**TTGTAAAACGACGGCCAGTG**GACAGCCCACACCTCATCAA	BS *hisD*
A426_pgi_leftflank_fw	**CCTGCAGGTCGACTCTAGAG**CAGCGGAATCGGCTGGTTG	Left flank for
A427_pgi_leftflank_rv	TCTTCTCTCATCCGCCAAAAGGTGCGTTTATTGGGCTGCT	BS *pgi*
A428_pgi_rightflank_fw	TGAACTTTAAGAAGGAGATATCATATGGCGGACATTTCGACCAC	Right flank for
A429_pgi_rightflank_rv	**TTGTAAAACGACGGCCAGTG**GCTGAGATACCAGCGGTCGC	BS *pgi*
A434_aceE_leftflank_fw	**CCTGCAGGTCGACTCTAGAG**CATGGATTCAACATGAAACCGC	Left flank for
A435_aceE_leftflank_rv	TCTTCTCTCATCCGCCAAAACACGCCGAAGTGCACGAATG	BS *aceE*
A436_aceE_rightflank_fw	TGAACTTTAAGAAGGAGATATCATATGGCCGATCAAGCAAAACTTG	Right flank for
A437_aceE_rightflank_rv	**TTGTAAAACGACGGCCAGTG**CCTCCATGAATGCACGTGCG	BS *aceE*
M19	CGCCAGGGTTTTCCCAGTCAC	Sequencing around MCS of pK19 plasmids
M20	AGCGGATAACAATTTCACACAGGA	
leftflank_cydD_del_fw	**CAGGTCGACTCTAGAGGATC**CTGGCATGGTCAACGTCTTC	Left flank for
leftflank_cydD_del_rv	GTCTGTAACCGAGCATCTCTCCTAGCGCAAAAGCCGCGGATCGAC	Δ*cydD*
rightflank_cydD_del_fw	GAGAGATGCTCGGTTACAGACTCCTGTTGGATGAGCCGAC	Right flank for
rightflank_cydD_del_rv	**GTAAAACGACGGCCAGTGAATTG**GACATCGGCAACCGAATCGA	Δ*cydD*
cydD-seq fw	TCTACCAAGGCTGGACCTAC	Sequencing
cydD-seq rv	CATCACTGGCGGCATTTCCC	*cydD* region
A156-ddh-qPCR-fw	CCGGAAAGCAAACCCACAAG	Housekeeping gene for normalization in qPCR (Frunzke et al., 2008)
A157-ddh-qPCR-rv	CTCGGAGTCGAAGGTTGCTT	
A269-hrtB-qPCR-fw	TCCGATTTAGCCTCACTCGC	Gene of interest for qPCR (Krüger and Frunzke, 2022)
A270-hrtB-qPCR-rv	AGTGACATCTGTTCGCCCTG	

Coloring scheme: overlap terminator, overlap terminator, Stop, RBS and linker, **overlap backbone,** overlap linker; BS = biosensor ::P_{hrtB}, MCS = multiple cloning site.

Table S2: Mutations established from ALE experiments with *C. glutamicum* ::P_{hrtB}_*pfkA* and ::P_{hrtB}_*aceE*

Clone	Mutation*	Gene	Annotation
*pfkA*1, *pfkA*2	Exchange A276V	NCgl2410	putative sugar diacid utilization transcriptional regulator
pfkA3	R307H	NCgl2276	Xanthine permease / 2-oxo-4-hydroxy-4-carboxy-5-ureidoimidazoline (OHCU) decarboxylase (EC 4.1.1.97)
pfkA3, pfkA4, pfkA5	A276V	NCgl2410	hypothetical protein
pfkA4	V358G	NCgl0322	5'-nucleotidase (EC 3.1.3.5)
pfkA5	G68S	NCgl0468	LSU ribosomal protein L10p (P0)
*pfkA*6, *pfkA*7, *pfkA*8	Exchange S164P	*miaB* (NCgl1874)	tRNA-i(6)A37 methylthiotransferase (EC 2.8.4.3)
*pfkA*6, *pfkA*7, *pfkA*8	Exchange C to T	Intergenic	
*pfkA*6, *pfkA*7, *pfkA*8	Deletion GCCCCCCGCG to GCCCCCGCG	Intergenic	
*pfkA*6, *pfkA*7, *pfkA*8	Exchange D385N	*benR* (NCgl2324)	Putative transcription regulator
*pfkA*6, *pfkA*7, *pfkA*8	Exchange A276V	NCgl2410	putative sugar diacid utilization transcriptional regulator
*pfkA*6, *pfkA*7, *pfkA*8	Exchange E305D	*wzz* (NCgl0337)	Tyrosine-protein kinase (EC 2.7.10.2)
*pfkA*6, *pfkA*7, *pfkA*8	Deletion E189fs	NCgl0627	2-methylcitrate dehydratase (EC 4.2.1.79)
*pfkA*7	Exchange Y91S	NCgl0919	hypothetical protein
*pfkA*7	Exchange L854R	NCgl1737	putative membrane protein CGP3 region
*pfkA*8	Exchange L184P	*bioQ* (NCgl2025)	Predicted biotin repressor from TetR family
aceE3, aceE 4	Exchange GTGTGGAA to ATATCAT	Intergenic	hypothetical protein
*aceE*1	Exchange R350C	NCgl1012	putative Mg-chelatase subunit ChlI, Inorganic ion transport, metabolism, and storage
*aceE*1	Exchange S70F	*ssuB* (NCgl1175)	aliphatic sulfonates ATP-binding ABC transporterprotein, essential for uptake of alkylsulfonates
*aceE*1	Exchange E122K	*gpsA* (NCgl1266)	NADPH-dependent glycerol-3-phosphate dehydrogenase
*aceE*1	Exchange S362N	NCgl1687	puative MoxR-like ATPase CGP3 region, Prophage gene
*aceE*1	Exchange R80H	*phoU* (NCgl2482)	phosphate uptake regulator
*aceE*1	Exchange V97A	*ispD* (NCgl2570)	2-C-methyl-D-erythritol 4-phosphate cytidylyltransferase
*aceE*1	Exchange A1910T	NCgl0705	Putative helicase

*= As identified by BV-BRC web resources (3.34.11)

References

Frunzke, J., Bramkamp, M., Schweitzer, J.E., and Bott, M. (2008). Population Heterogeneity in *Corynebacterium glutamicum* ATCC 13032 caused by prophage CGP3. J Bacteriol *190*, 5111-5119.

Heyer, A., Gätgens, C., Hentschel, E., Kalinowski, J., Bott, M., and Frunzke, J. (2012). The two-component system ChrSA is crucial for haem tolerance and interferes with HrrSA in haem-dependent gene regulation in *Corynebacterium glutamicum*. Microbiology (Reading) *158*, 3020-3031.

Krüger, A., and Frunzke, J. (2022). A pseudokinase version of the histidine kinase ChrS promotes high heme tolerance of *Corynebacterium glutamicum*. Front Microbiol *13*, 997448.

Livak, K.J., and Schmittgen, T.D. (2001). Analysis of relative gene expression data using real-time quantitative PCR and the 2(-Delta Delta C(T)) Method. Methods *25*, 402-408.
